# Unmet Health Care Needs Among Young Transgender Women at Risk for HIV Transmission and Acquisition in Two Urban U.S. Cities: The LifeSkills Study

**DOI:** 10.1089/trgh.2018.0026

**Published:** 2019-01-17

**Authors:** John Frank, Arjee Restar, Lisa Kuhns, Sari Reisner, Katie Biello, Robert Garofalo, Matthew J. Mimiaga

**Affiliations:** ^1^ Department of Behavioral and Social Health Sciences, Brown University School of Public Health, Providence, Rhode Island.; ^2^ Department of Epidemiology, Brown University School of Public Health, Providence, Rhode Island.; ^3^ Center for Health Equity Research, Brown University, Providence, Rhode Island.; ^4^ Department of Psychiatry and Human Behavior, Alpert Medical School, Brown University, Providence, Rhode Island.; ^5^ Division of Adolescent Medicine, Ann & Robert H. Lurie Children's Hospital of Chicago, Chicago, Illinois.; ^6^ Department of Pediatrics, Feinberg School of Medicine, Northwestern University, Chicago, Illinois.; ^7^ The Fenway Institute, Fenway Health, Boston, Massachusetts.; ^8^ Division of General Pediatrics, Boston Children's Hospital/Harvard Medical School, Boston, Massachusetts.; ^9^ Department of Epidemiology, Harvard T.H. Chan School of Public Health, Boston, Massachusetts.

**Keywords:** access to care, HIV, transgender women, young adults

## Abstract

**Purpose:** The physical health care needs of transgender women are not being adequately addressed in the United States. The current study adds to the literature on the state of health care among young transgender women (YTW) by describing the occurrence of unmet health needs among a sample of YTW and providing unique data on psychosocial and demographic factors associated with access to adequate care.

**Methods:** Baseline data were analyzed from Project LifeSkills, an intervention study funded by the National Institutes of Health (NIH). YTW (*N*=300) between the ages of 16 and 29 were recruited from the Boston and Chicago metropolitan areas between 2012 and 2015. Data were collected on health care experiences, indicators of social marginalization, and sociodemographic information. The final analytic sample (*N*=273) was restricted to participants with complete data; participants that were removed did not significantly differ demographically from the final analytic sample retained. Bivariate logistic regression models examined the association between having unmet health care needs and sociodemographics, social marginalization, and health care utilization indicators. A final adjusted multivariable logistic regression model was constructed with independent variables that were statistically significant in bivariate models.

**Results:** Overall, nearly a quarter (23%) of YTW indicated that they had unmet health care needs. In the final multivariable model adjusted for enrollment city, avoiding health care due to cost (adjusted odds ratio [aOR]=1.98, 95% confidence interval [CI]=1.05–3.76) and experiencing prior transgender-specific discrimination in a medical setting (aOR=4.54, 95% CI=2.30–8.95]) were associated with a greater odds of having unmet health care needs.

**Conclusion:** YTW face significant barriers to accessing health care in the United States. Among this sample, prior experiences of discrimination and inability to afford health care increased YTW odds of having unmet health care needs. Efforts to improve the unmet health care needs among YTW should promote access to affordable, gender-affirming care.

## Introduction

Transgender is a term used to describe people whose gender identity, expression, or behavior differs from “those typically associated with their assigned sex at birth.”^1^ In the United States, transgender people face elevated rates of discrimination, harassment, stigma, and violence across multiple settings, that is, family, school, workplace, and public accommodations,^2,3^ which contribute to financial instability,^4^ difficulties attaining housing,^4^ lower levels of educational attainment,^4^ and adverse mental and physical health outcomes.^4–9^ Compared with their cisgender (i.e., people whose gender identity matches their assigned sex at birth) peers, transgender people report higher rates of depression,^4,5,7,8^ anxiety,^4^ HIV/sexually transmitted infections (STIs),^10^ smoking,^8^ binge drinking,^8^ and poor overall health.^4,8^

The elevated health care needs faced by transgender people are exacerbated by disparities in access to treatment.^9,11,12^ Although limited research exists on the state of health care for transgender people living in the United States, recent findings indicate that transgender individuals face multiple barriers to routine, preventive, and emergency health care.^9,11,12^ Results of the 2015 U.S. Transgender Survey (USTS), the largest study of transgender people in the United States (*N*=27,715), reveal that 33% of participants were unable to attain care when it was needed.^12^ In the National Transgender Discrimination Study (NTDS), a survey of 6,450 transgender adults from across the United States, 48% of participants delayed medical care when they were injured or sick due to cost.^11^

Among a community-based sample of 452 transgender and gender-nonconforming people living in Massachusetts, 19% of participants avoided care when sick or injured and 24% of participants delayed routine preventative care.^9^ Furthermore, transgender women (i.e., transgender individuals who identify as women, male-to-female, or another transfeminine identity)^13^ are disproportionally affected by HIV,^14^ and barriers to health care access interfere with both HIV treatment^13,15^ and HIV prevention strategies (i.e., pre-exposure prophylaxis).^13^

Researchers are beginning to identify barriers that contribute to the disparities in access to health care among transgender individuals. This includes examining health care indicators, such as prior discrimination and anticipated discrimination in health care settings, limited availability of competent providers, and cost of care.^3,9,16–20^ Participants in a qualitative study of 30 transgender and gender-nonconforming individuals noted that they anticipate rejection and discriminatory treatment from health care providers and will avoid health care due to anticipated discrimination.^17^ An association between discrimination and health care access has also been identified in quantitative studies. In Massachusetts, prior experience with discrimination both in health care settings and nonhealth care settings (e.g., retail, restaurants, and transportation) was associated with delaying routine preventive care, treatment for sickness or injury, and emergency care among transgender and gender-nonconforming adults.^3^ In the USTS, 33% of transgender participants who saw a provider in the past year experienced discrimination in that setting, and 23% of all participants avoided seeing a doctor when they needed care due to anticipated discrimination.^12^ Anticipated stigma was also associated with lower utilization of health services and worse overall health in a sample of rural transgender adults.^8^ Among Lesbian, Gay, Bisexual, Transgender, and Questioning (LGBTQ) emerging adults, transgender participants were more likely to report that their relationship with their provider was negatively impacted by disclosing their LGBTQ identity than their lesbian, gay, or bisexual cisgender peers.^16^ As a consequence, transgender emerging adults were also more likely to delay care due to LGBTQ-related discrimination.^16^ When seeking gender-affirming health care, transgender youth and their caregivers reported difficulty finding competent providers and noted that the youth faced discrimination by providers (i.e., inconsistent use of chosen name/pronoun).^21^ Finally, 33% of participants in the USTS sample of 27,715 transgender adults avoided health care because they could not afford it.^12^

More research is needed to address the unique physical health care needs of young transgender women (YTW).^22,23^ Researchers are beginning to identify barriers to care,^21–23^ but continued research is needed to understand their impact among YTW. The current study provides unique data on YTW's sociodemographic and health care factors associated with unmet health care needs. Given the existing research on barriers to health care utilization,^3,9,16–20^ we hypothesized that having unmet health care needs was associated with lack of relationship to a primary care provider, having government-issued insurance, avoiding care due to cost, past experience of discrimination in health care settings, and not having had a routine checkup within the past year.

## Methods

### Study participants and procedures

Study description and procedures have been thoroughly described elsewhere.^24^ Briefly, between 2012 and 2015, 300 sexually active YTW, 16 through 29 years of age, enrolled in Project LifeSkills, a randomized controlled efficacy trial of a behavioral HIV prevention intervention in Chicago and Boston. Participant recruitment and data collection used principles based on community-based participatory research, that is, research teams from both sites consisted of community members, including YTW who collaborated on efforts to identify locations where YTW gather, recruit participants for the study, and conduct study assessments with participants. Participants were recruited using convenience sampling methodologies. Recruitment sites included bar/nightclubs, community centers, and online through Craigslist and Facebook. Eligibility criteria were: between ages of 16 and 29, identify along the transfeminine spectrum (e.g., trans woman, male-to-female, female) and assigned male sex at birth, able to speak and understand English, and reported engaging in risky sexual behavior, that is, condomless anal or vaginal intercourse, anal or vaginal intercourse with more than one sexual partner, anal or vaginal sex in exchange of money, food, shelter, or prior diagnosis of HIV or another STI. Participants completed a 2-h quantitative assessment using computer-assisted self-interviewing (ACASI). Written consent was obtained from all eligible participants. All study procedures were approved by Institutional Review Committees at both participating sites.

### Measures

Data utilized for this analysis were from baseline measures of sociodemographics, social marginalization, and health care utilization, and are detailed below.

#### Sociodemographics

To assess for sociodemographic information, we asked participants about their age (categorized into 16–20, 21–25, and 26–29), race, and ethnicity (Black, Latina, White, other race/ethnicity), income (dichotomized into <10,000 USD vs. 10,000 USD or more), current employment status (yes vs. no), self-reported sexual orientation (lesbian, gay, bisexual, heterosexual, other sexual orientation/not listed), education attained (dichotomized to high school or less vs. college or more), and self-reported HIV status (positive, negative, or unknown). Of note, participants were not asked to report the gender identity of their sexual partners, and self-reported sexual orientation was based on participants' understanding of the options listed.

#### Social marginalization

Social marginalization indicators included asking participants about their history of having engaged in sex work in the past 4 months (yes vs. no), experienced homelessness in the past 4 months (yes vs. no), and spent time in jail or experienced incarceration within the past 4 months (yes vs. no).

#### Health care utilization experiences

We used a total of six items to measure health care utilization. This includes asking participants about having government-issued insurance (yes vs. no), having a relationship with at least one primary care provider (yes vs.no), avoiding health care due to cost (yes vs. no), having last health checkup within the past year (yes vs. no), and having faced discrimination in medical setting due to their transgender identity (yes vs. no).

#### Primary outcome

Our primary outcome is frequency of unmet health needs of YTW. We assessed this outcome by asking: “How often do your health care services meet your specific health needs?” Responses where dichotomized to either: always/usually versus not always/not usually.

### Analyses plan

The final analytic sample (*N*=273) was restricted to participants with complete data; participants that were removed did not significantly differ demographically from the final analytic sample retained. Descriptive statistics were calculated for sociodemographics, social marginalization, and health care utilization indicators. Logistic regression was used to examine bivariate associations of having unmet health care needs with sociodemographics, social marginalization, and health care utilization indicators. A final multivariable logistic regression model was constructed with the same outcome and included the independent variables that were statistically significant in the bivariate models. All analyses were conducted using SPSS statistical package, version 23.0,^25^ with alpha set to <0.05 *a priori*.

## Results

Table 1 displays the examined characteristics of sociodemographics, social marginalization, and health care utilization experiences. For our primary outcome, nearly one-fourth (23%) reported having unmet health care needs.

**Table 1.** Bivariate Logistic Regressions Modeling Young Transgender Women's (*N*=273) Unmet Health Needs with Associated Characteristics of Sociodemographics, Social Marginalized, and Health Care Utilization Experiences

	*n* (%)	Unmet health needs, *n* (%)	Met health needs, *n* (%)	χ^2^*p*-value
Total	273 (100.0)	63 (23.1)	210 (76.9)	
Study site				
Boston	140 (51.3)	26 (18.6)	114 (81.4)	0.070
Chicago	133 (48.7)	37 (27.8)	96 (72.2)	
Sociodemographics				
Age				
16–20	51 (18.7)	9 (17.6)	42 (82.4)	0.236
21–25	135 (49.5)	37 (27.4)	98 (72.6)	
26–29	87 (31.9)	17 (19.5)	70 (80.5)	
Race/ethnicity				
Black	129 (47.3)	30 (23.3)	99 (76.7)	0.954
Latina/Hispanic	37 (13.6)	9 (24.3)	28 (75.7)	
White	71 (26.0)	17 (23.9)	54 (76.1)	
Other race/ethnicity	36 (13.2)	7 (19.4)	29 (80.6)	
Currently employed				
Yes	72 (26.4)	13 (18.1)	59 (81.9)	0.239
No	201 (73.6)	50 (24.9)	151 (75.1)	
Income				
<10K	128 (46.9)	35 (27.3)	93 (72.7)	0.116
>10K	145 (53.1)	28 (19.3)	117 (80.7)	
Sexual orientation				
Gay	68 (24.9)	17 (25.0)	51 (75.0)	0.880
Lesbian	15 (5.5)	2 (13.3)	12 (86.7)	
Bisexual	53 (19.4)	12 (2.6)	41 (77.4)	
Other sexual orientation/not listed	26 (9.5)	7 (26.9)	19 (73.1)	
Heterosexual	111 (40.7)	25 (22.5)	86 (77.5)	
Education				
High school or less	169 (61.9)	34 (20.1)	135 (79.9)	0.139
College or more	104 (38.1)	29 (27.9)	75 (72.1)	
HIV status				
Positive	58 (21.3)	10 (17.2)	48 (82.8)	0.463
Negative	212 (77.7)	52 (24.5)	160 (75.5)	
Unknown	3 (1.1)	1 (33.3)	2 (66.7)	
Social marginalization				
Sex work engagement (<4 months)				
Yes	98 (35.9)	23 (23.5)	75 (76.5)	0.908
No	175 (64.1)	40 (22.9)	135 (77.1)	
Recent homelessness (<4 months)				
Yes	63 (23.1)	17 (27.0)	46 (73.0)	0.401
No	210 (76.9)	46 (21.9)	164 (78.1)	
Jail/incarceration				
Yes	22 (8.1)	6 (27.3)	16 (72.7)	0.626
No	251 (91.9)	57 (22.7)	194 (77.3)	
Health care experience				
Relationship with at least one PCP				
Yes	208 (76.2)	42 (20.2)	166 (79.8)	0.043^a^
No	65 (23.8)	21 (32.3)	44 (67.7)	
Government-based insurance				
Yes	171 (62.6)	32 (18.7)	139 (81.3)	0.027^a^
No	102 (37.4)	31 (30.4)	71 (69.6)	
Avoided health care due to cost				
Yes	198 (72.5)	35 (17.7)	163 (82.3)	0.001^b^
No	75 (27.5)	28 (37.3)	47 (62.7)	
Had last checkup within past year				
Yes	228 (83.5)	48 (21.1)	180 (78.9)	0.074
No	45 (16.5)	15 (33.3)	30 (66.7)	
Face discrimination in medical setting due to transgender identity				
Yes	54 (19.8)	26 (48.1)	28 (51.9)	0.000^c^
No	219 (80.2)	37 (16.9)	182 (83.1)	

^a^ *p*<0.05; ^b^*p*<0.01; ^c^*p*<0.001.

### Sociodemographics

Half (50%) of the sample was between 21 and 25 years of age (mean=23.4 years old, standard deviation=3.5 years old). The sample was racially/ethnically diverse, with 47% Black, 13% Latina, 26% White, and 13% other race/ethnicity. About three-fourths (73%) were unemployed, and almost half (47%) were below poverty line, earning <10K. There was heterogeneity with sexuality orientation, with largest portion identifying as heterosexual (41%), followed by gay (25%), bisexual (19%), other sexual orientation/not listed (10%), and lesbian (5%). Most YTW in this sample attained education of high school or less (62%). More than a fifth (21%) were HIV positive.

### Social marginalization

The prevalence of recent engagement (<4 months) with sex work was ∼36% percent of the sample. Almost a quarter (23%) of participants recently experienced homelessness, and 8% were recently jailed/incarcerated.

### Health care utilization experiences

Indicators of health care utilization show that there is a high proportion of YTW in this sample with government-based issued insurance (63%) and who had a medical checkup within the past year (84%). However, approximately one quarter (24%) of participants reported not having a relationship with at least one primary care provider (24%). Furthermore, about a third (28%) avoided health care due to cost, and a fifth (20%) faced discrimination in medical setting due to transgender identity.

### Regression models

With the exception to having had medical checkup within the past year, all health care utilization indicators were significantly associated with unmet health needs (Table 2).

**Table 2.** Final Logistic Regression Model of Young Transgender Women's (*N*=273) Unmet Health Needs with Independently Associated Health Care Utilization Factors (Adjusted for Study Site)

	OR (95% CI)	*p*	aOR (95% CI)	*p*
Total	273 (100.0)			
Primary predictors				
Relationship with at least one PCP				
Yes	0.52 (0.28–0.99)	0.04^a^	0.68 (0.32–1.43)	0.13
No	Ref.		Ref.	
Government-based insurance				
Yes	0.53 (0.30–0.93)	0.03^a^	0.70 (0.36–1.34)	0.20
No	Ref.		Ref.	
Avoided health care due to cost				
Yes	2.77 (1.53–5.02)	0.00^b^	1.98 (1.05–3.76)	0.03^a^
No	Ref.		Ref.	
Face discrimination in medical setting due to transgender identity				
Yes	4.56 (2.40–8.66)	0.00^b^	4.54 (2.30–8.95)	0.00^b^
No	Ref.		Ref.	

^a^ *p*<0.05; ^b^*p*<0.01.

aOR, adjusted odds ratio; CI, confidence interval; OR, odds ratio.

In our bivariate unadjusted model, having a relationship with at least one primary care provider and having government-based insurance were protective factors to having unmet health needs. Specifically, YTW who reported having a relationship with at least one primary care provider (odds ratio [OR]=0.52, 95% confidence interval [CI]=0.28–0.99) had significantly lower odds of having unmet health needs. YTW who reported having a government-based insurance (OR=0.53, 95% CI=0.30–0.93) had significantly lower odds of having unmet health needs.

On the other hand, having avoided health care due to cost and experienced facing discrimination in medical setting due to transgender identity were risk factors for unmet health needs. YTW who reported having avoided health care due to cost (OR=2.77, 95% CI=1.53–5.02) had significantly higher odds of having unmet health needs. In addition, YTW who reported having experienced facing discrimination in a medical setting due to transgender identity (OR=4.56, 95% CI=2.40–8.66) had significantly higher odds of having unmet health needs.

In the final multivariable model adjusted for study site, participants who avoided health care due to cost (adjusted odds ratio [aOR]=1.98, 95% CI=1.05–3.76) and those who had experienced prior discrimination in medical settings due to their transgender identity (aOR=4.54, 95% CI=2.30–8.95) had greater odds of having unmet health care needs. However, having a relationship with at least one primary care provider and having government-based insurance were no longer significantly associated with YTW's unmet health needs.

## Discussion

The current study provides data on the state of health care utilization and factors associated with unmet health care needs among YTW from two urban cities, Boston and Chicago, in the United States. Our findings show that YTW face several barriers in accessing health care (e.g., not having health insurance coverage, anticipated cost of care) as well as negative experiences when in care (e.g., discrimination due to being transgender, not having relationships with primary care provider). In this sample, YTW reported not having health insurance coverage more than twice the rate of the U.S. general population (21.6% vs. 9.1%, respectively).^26^ Over a quarter of YTW (28%) in this sample indicated that they had avoided seeking out health care due to the anticipated cost, which is relatively similar to the rate reported by the USTS (33%). These findings support previous studies that documented multiple barriers and negative experiences among transgender populations in health care settings.^3,11,12,15,27–29^ Our findings highlight that although majority of YTW in this sample are accessing and utilizing health care, there remains unmet health needs specific to this group. Nearly one-quarter (23%) of the YTW in the study indicated that they had health care needs that were not adequately addressed. This is concerning given that there was a high proportion of YTW participants in this sample that had access to medical checkup within the past year (84%). This suggests that while many YTW are connected to the health care system and are receiving some form of medical care, these services most likely are not meeting all of YTW's health needs.

Furthermore, participants who avoided health care due to cost reported significantly greater unmet health needs. Specifically, the odds of having unmet health care needs for those who avoided health care due to cost was almost twice the odds of those who did not avoid. This indicates that for YTW, financial circumstances play a role in their avoidance of health care and the kinds of health care services that are meeting their health needs.^19,30–32^ Despite that many of YTW participants in this study have government-issued insurance (63%), this finding suggests that YTW continue to experience barriers related to cost when it comes to meeting their health care needs. Previous studies examining the impact of federal-level policies on heath insurances have noted insufficient medical coverage for transgender-related health services that contradicts current standards of medical care for transgender people, and those that do cover transgender-related health services are poorly marketed to transgender populations.^18,33^ As such, future research should look at how cost and limited insurance coverage for transgender-related health services may play a role in preventing YTW's health care needs from being met. To address the unmet health needs of YTW, it is important for YTW to be linked with health insurance that adequately covers their specified health needs.

We also found significantly greater unmet health needs among YTW who reported experiencing discrimination in medical setting due to their transgender identity. Among those who experienced discrimination in medical settings due to their transgender identity, the odds of having unmet health needs were greater than four times compared with those who did not experience discrimination. We found that three in four YTW in this sample did not have a relationship with a primary care provider. However, access to a primary care provider is insufficient for addressing health care needs for YTW, as past studies have shown that among health care providers who have interacted with transgender patients have concerns about being incompetent due to poor knowledge of transgender health and lack the interpersonal skills necessary for interacting and discussing sexual, physical, and mental health with transgender patients, which contribute to provider's uncertainties of delivering care.^20,34^ While such provider-level issues are likely to contribute to YTW's unmet health care needs, our study points that there could also be lack of structural or institutional services and resources for YTW that are not integrated in health care systems. For example, Reisner et al.^35^ emphasized that health care settings which facilitate gender-affirmative services and provider–patient interactions (i.e., provision of accessible, holistic, and affirming health care services that is optimally and culturally tailored to transgender people's health needs) are vital for YTW's engagement in health care. Further research that elicits and addresses unmet health needs for YTW is necessary to address gaps in health care provision among this group.

### Limitations

In our final model, there was no association between having a relationship with a primary care provider (PCP) and unmet health care needs. The lack of an association between these experiences may be due to the considerable proportion (24%) of YTW in the sample who reported not having a relationship with at least one PCP. Additionally, the impact of having a PCP on unmet health care needs may have been masked by the relationship between avoiding health care due to cost and having a relationship with PCP. It is possible that financial considerations prevent YTW from establishing relationships with PCPs, and lack of access to a PCP mediates the relationship between avoidance due to cost and unmet needs. Future longitudinal analyses should further examine the interrelations of these variables. Moreover, because data for this study comes from an HIV prevention intervention with a different aim seeking to lower risk behaviors among YTW, the lack of associations between our independent variables and our main outcome in the current analysis may be due to not having collected detailed measures to power on our main research question.

The current analyses were conducted on baseline data from an HIV prevention study targeting YTW who engaged in high-risk behavior.^24^ Eligibility criteria for the parent study may limit the extent to which the current findings generalize to the population of YTW in the United States. Additionally, YTW who agreed to participate in the parent study are likely motivated to engage in health-promoting behavior and, thus, the impact of barriers to care may be attenuated among this population. The parent study employed a form of convenience sampling in which existing community members helped identify study participants, and findings may not reflect the experiences of YTW who are less connected to existing networks of YTW in the two urban cities from which the sample was recruited. Additional barriers to health care may exist for YTW who are less connected to existing networks. Analyses were conducted on cross-sectional data and do not provide evidence for a causal relationship between the identified health care barriers and unmet health care needs.

### Public health implications

Taken together, our findings point to the importance of both health care access (e.g., having health insurance with adequate coverage, having positive relationships with health care providers) and health care services and settings (e.g., connecting YTW to facilities with gender affirmative services and nondiscriminatory practices) in addressing unmet health needs of YTW. Future studies should further investigate aspects of provider–patient relationship and the kinds of services specific to YTW's health needs are lacking or not being met within primary care settings, including cost and insurance. Policies relating to insurance coverage, cost of services, and antidiscrimination specific to transgender identities should be visible or conspicuous to YTW seeking health services to alleviate perceptions of cost and discriminatory care when inviting YTW to engage in health care settings.

## Ethics Approval

All procedures performed in studies involving human participants were in accordance with the ethical standards of the Institutional Review Boards of The Fenway Institute (Boston, MA), Ann & Robert H. Lurie Children's Hospital (Chicago, IL), and with the 1964 Helsinki declaration and its later amendments or comparable ethical standards.

## Informed Consent

Informed consent was obtained from all individual participants included in the study.

## Abbreviations Used

aOR: adjusted odds ratio
CI: confidence interval
NIH: National Institutes of Health
OR: odds ratio
USTS: U.S. Transgender Survey
YTW: young transgender women
